# Let-7a-5p regulates the inflammatory response in chronic rhinosinusitis with nasal polyps

**DOI:** 10.1186/s13000-021-01089-0

**Published:** 2021-03-30

**Authors:** Jianwei Zhang, Lei Han, Feng Chen

**Affiliations:** 1 Department of Otolaryngology-Head and Neck Surgery, Shanghai Pudong New District Gongli Hospital/Second Military Medical University Affiliated Hospital, Shanghai, 200135 China; 2 Department of Otolaryngology-Head and Neck Surgery, Suqian First People’s Hospital, The Suqian Clinical college of Xuzhou Medical University, Suqian, 223800 Jiangsu Province China; 3 Department of Otorhinolaryngology-Head and Neck Surgery/ Research Institution of Otorhinolaryngology, Nanjing Drum Tower Hospital (The Affiliated Hospital of Nanjing University Medical School), Nanjing, 210008 Jiangsu Province China

**Keywords:** Chronic rhinosinusitis with nasal polyps, Inflammatory response, IL-6, Let-7a-5p, Ras-MAPK

## Abstract

**Background:**

Let-7a-5p is demonstrated to be a tumor inhibitor in nasopharyngeal carcinoma. However, the role of let-7a-5p in chronic rhinosinusitis with nasal polyps (CRSwNP) has not been reported. This study is designed to determine the pattern of expression and role of let-7a-5p in CRSwNP.

**Methods:**

The expression level of let-7a-5p, TNF-α, IL-1β, and IL-6 in CRSwNP tissues and cells were detected by RT-qPCR. Western blot assay was carried out to measure the protein expression of the Ras-MAPK pathway. Dual luciferase reporter assay and RNA pull-down assay were used to explore the relationship between let-7a-5p and IL-6.

**Results:**

Let-7a-5p was significantly downregulated in CRSwNP tissues and cells. Moreover, the mRNA expression of TNF-α, IL-1β and IL-6 was increased in CRSwNP tissues, while let-7a-5p mimic inhibited the expression of TNF-α, IL-1β and IL-6. Besides that, let-7a-5p was negatively correlated with TNF-α, IL-1β and IL-6 in CRSwNP tissues. In our study, IL-6 was found to be a target gene of let-7a-5p. Additionally, let-7-5p mimic obviously reduced the protein levels of Ras, p-Raf1, p-MEK1 and p-ERK1/2, while IL-6 overexpression destroyed the inhibitory effect of let-7a-5p on the Ras-MAPK pathway in CRSwNP.

**Conclusion:**

We demonstrated that let-7a-5p/IL-6 interaction regulated the inflammatory response through the Ras-MAPK pathway in CRSwNP.

## Introduction

Chronic rhinosinusitis (CRS) is one of the common chronic diseases in otorhinolaryngology, and its incidence rate is increasing year by year [1]. At present, CRS is divided into two types: CRS with nasal polyps (CRSwNP) and CRS without nasal polyps (CRSsNP) [2]. CRSwNP is characterized by eosinophilic inflammation of the nasal-sinus mucosa [3]. The main symptoms include nasal congestion, sticky or purulent nasal discharge, headache, and hyposmia or loss of sense of smell [4]. The chronic recurrent CRSwNP seriously affects the patient’s quality of life and brings a heavy economic burden. Some cases of refractory CRSwNP cannot be reversed by surgery or antimicrobial therapy, which is also a very challenging situation for both patients and doctors [5]. It is currently believed that the inflammatory response of Th2 cells caused by microbial stimulation is the main cause of CRSwNP [6]. However, no specific pathogens or exogenous stimuli were directly associated with the development of CRSwNP. Although many studies have been conducted on the role of chronic inflammation in the development of CRSwNP, the underlying mechanism remains unclear.

MicroRNA (miRNA) is a type of endogenous non-coding single-stranded small RNA with a length of about 22 nt [7]. It can regulate the expression of target genes after transcription by cutting the transcription products or inhibiting the translation of the transcription products [8, 9]. MiRNAs are widely involved in various of biological events and the pathogenesis of a variety of diseases [10]. It was found that miR-125b was involved in the pathogenesis of acidophilic CRSwNP by suppressing 4E-BP1expression [11]. Moreover, Liu et al. discovered that miR-124 participated in the regulation of inflammatory response by mediating the expression of AHR in CRSwNP [12]. Previous studies have demonstrated that let-7a-5p is down expressed in various human tumors [13]. For example, let-7a-5p was found to suppress cell proliferation, migration and invasion in triple-negative breast cancer [14]. Moreover, let-7a-5p inhibited cell growth, metastasis and the doxorubicin resistance in prostate cancer [15]. However, the role of let-7a-5p in CRSwNP has not been elaborated.

In this study, we detected the expression pattern of let-7a-5p in CRSwNP, and explored its effect on inflammatory factors. Besides that, we demonstrated that let-7a-5p regulated the **i**nflammatory response by modulating IL-6 expression in CRSwNP.

## Materials and methods

### Clinical samples

Twenty CRSwNP tissues and normal nasal mucosa tissues were collected from patients with CRSwNP from 2018 to 2019 at Shanghai Pudong New District Gongli Hospital/ Second Military Medical University Affiliated Hospital. Among the 20 tissues, 14 cases are edema type, 4 cases are glandular hyperplasia type, and 2 cases are fibrous type. CRSwNP tissues were collected from nasal polyps in the middle nasal meatus of patients, and normal nasal mucosa tissues were collected from the inferior turbinate of patients. All patients had no history of allergies, asthma, or aspirin sensitivity within 8 weeks before the biopsy, and did not receive topical or systemic steroids, non-steroidal anti-inflammatory drugs, antihistamines or antibiotics. The general information of 20 patients with CRSwNP is shown in Table 1. This study was approved by the Medical Ethics Committee of Shanghai Pudong New District Gongli Hospital/ Second Military Medical University Affiliated Hospital. Each patient signed the informed consent.

**Table 1:** Clinical characteristics of CRSwNP patients

	CRSwNP
Sample number	20
Mean age (years)	50 ± 3.5
Male %	12 (60%)
Female%	8 (40%)
Allergy (%)	10 (50%)
Asthma (%)	5 (25%)
CT score	19 (14–21)
Endoscopic score	2 (2–3)
Asthma comorbidity	0
Aspirin intolerance	0

*CRSwNP* Chronic rhinosinusitis with nasal polyps

### Cell culture

NorDFs cells were isolated from surgical tissues, and NPDFs cells were obtained from patients undergoing CRSwNP endoscopic sinus surgery. Cells were cultured with Dulbecco’s Modified Eagle Medium (DMEM) with 10% Foetal Bovine Serum (FBS), 10,000 mg/ml penicillin and 10,000 mg/ml streptomycin. Cells were trypsinized with 0.05% Ethylene Diamine Tetraacetic Acid (EDTA). The purity of NorDFs and NPDFs cells was confirmed microscopically by their characteristic spindle cell morphology.

### Cell transfection

Cells were cultured in 6-well plates. After 24 h of incubation, the cells were randomly divided into control group, miR-NC group, let-7a-5p mimic group, let-7a-5p mimic+ pcDNA3.1-IL-6 group. Let-7a-5p mimic and miR-NC were purchased from GenePharma (Shanghai, China). The full-length sequences of IL-6 were respectively synthesized and cloned into pcDNA3.1 (Invitrogen, Carlsbad, USA) plasmid to produce pcDNA3.1-IL-6. Lipofectamine 3000 (L3000015, Thermo Fisher Scientific, USA) was used as a transfection reagent for cell transfection.

### RNA extraction and reverse transcription-quantitative (RT-q) PCR assay

Total RNA was extracted by TRIZOL reagent (Invitrogen, Thermo Fisher Scientific, Inc., USA). The cDNA template was synthesized by reverse transcription in the PCR Amplifier, and RT-qPCR was conducted with ABI7500 (Applied Biosystems, USA). The reaction conditions were initial denaturation at 95 °C for 10 min, followed by 40 cycles of denaturation at 95 °C for 10 s, annealing at 60 °C for 20 s, and extension at 72 °C for 34 s. The 2^-ΔΔCt^ method was used to analyze the experimental results. U6 was the endogenous control of let-7a-5p, while GAPDH was used as the endogenous control of IL-6, TNF-α, and IL-1β. The primer sequences were synthesized by Shanghai Sangon Bioengineering Co., LTD. (Shanghai, China), and were shown in Table 2.

**Table 2:** The primers sequences in qRT-PCR

Gene		Primers sequences
let-7a-5p	Forward	5′-GGGAGAAGTCCGCTGGTGTTG-3′
let-7a-5p	Reverse	5′-CTGATCTCCTTGTTCAAGTTCA-3′
U6	Forward	5′-CTCGCTTCGGCAGCACA-3′
U6	Reverse	5′-AACGCTTCACGAATTTGCGT-3′
TNF-α	Forward	5′-CACCATGAGCACTGAAAGCA-3′
TNF-α	Reverse	5′-GCTCTTGATGGCAGAGAGGAG-3′
IL-1β	Forward	5′-TTCGAGGCACAAGGCACAAC-3′
IL-1β	Reverse	5′-CTGGAAGGAGCACTTCATCTGT-3′
IL-6	Forward	5′-ACCCCCAGGAGAAGATTCCA-3′
IL-6	Reverse	5′-GTCTTCCCCCACACCAAGTT-3′
GAPDH	Forward	5′-GCCACAACGACCCCTTCATG-3′
GAPDH	Reverse	5′-TGCCAGTGAGCTTCCCGTTC-3′

### ELISA assay

ELISA assay was used to detect the levels of inflammatory cytokines IL-6, TNF-α and IL-1β. Venous blood (1 ml) was centrifuged at 3000 rpm for 10 min. Then the supernatant was collected in EP (eppendorf) tubes and stored at − 70 °C. The levels of IL-6, TNF-α, and IL-1β were tested by kits from R&D (R&D, USA) according to the manufacturer’s instructions. After incubation at room temperature for 2 h, and then incubated with horseradish peroxidase (200 μL) for another 2 h. Then, added 200 μL TMB-HCL and incubated in darkness at room temperature for 30 min. Finally, 50 μL H_2_SO_4_ (2 mol/L) was added to each well plate. After 30 min, the absorbance at 450 nm was determined with a microplate reader.

### Western blot assay

The protein concentration was measured by using the BCA kit (ThermoFisher Scientific, USA). The protein sample was added with 5 × Loading buffer, boiled in water for 10 min, and then carried out sodium dodecyl sulfate polyacrylamide gel electrophoresis (SDS-PAGE) protein electrophoresis. After the electrophoresis, the protein was transfected to the polyvinylidene difluoride (PVDF) membrane, and blocked with 5% skim milk for 2 h at room temperature. The membranes were incubated with specific antibodies (Rabbit anti-Human, anti-GAPDH, Ras, p-Raf1, Raf1, p-MEK1, MEK1, p-ERK1/2, ERK1/2, 1:1000, Abcam) overnight at 4 °C. Then, membranes were incubated with the horseradish peroxidase-conjugated antibody (Goat anti-Rabbit IgG, 1:1000, Sigma) at room temperature for 1 h. ECL reagent (Beyotime) was used to detect the protein signals.

### Dual luciferase reporter assay

The combination of let-7a-5p and IL-6 in the 3′-UTR region was predicted by Targetscan software. According to the predicted results, the mutant and wild sequences of let-7a-5p and IL-6 binding sites were designed, respectively. Mutant and wild sequence fragments were cloned and combined with the Promega vector. IL-6-Mut and IL-6-Wt were transfected with let-7a-5p mimic or miR-NC, respectively. Finally, luciferase activity was detected and normalized to the Renilla luciferase activity.

### RNA-pull down assay

Bio-IL-6-Wt, Bio-IL-6-Mut and Bio-NC were obtained from GenePharma (Shanghai, China). After transfection for 48 h, the cells were lysed with RIPA buffer (Beyotime, Shanghai, China). The cell lysate was incubated with Dynabeads M-280 Streptavidin (Invitrogen, CA). Finally, the enrichment of let-7a-5p was explored by RT-qPCR.

### Statistical analysis

SPSS 22.0 and GraphPad Prism 7.01 were used to analyze the data. All data were expressed as mean ± standard deviation (SD), and all experiments were repeated three times. Unpaired Student’s *t*-test was carried out to test the differences between the two groups. Moreover, multiple groups were compared using One-way ANOVA and Tukey’s test. *P* < 0.05 was considered statistically significant.

## Results

### Let-7a-5p was down regulated in CRSwNP tissues and cells

**Fig. 1:**
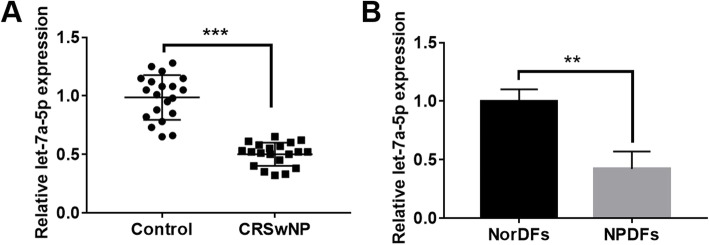
let-7a-5p was down regulated in CRSwNP tissues and cells. **a** The expression of let-7a-5p in CRSwNP tissues and control group was detected by RT-qPCR. **b** The expression of let-7a-5p was explored in NorDFs cells and NPDFs cells. ***p < 0.001

First, we explored the expression pattern of let-7a-5p in CRSwNP by qRT-PCR assay. The results displayed that let-7a-5p was obviously downregulated in CRSwNP tissues compared with normal group (*p* < 0.001; Fig. 1a). Similarly, let-7a-5p showed a downward trend in NPDFs cells compared with NorDFs cells (*p* < 0.001; Fig. 1b).

### Let-7a-5p expression was negatively correlated with TNF-α, IL-1βand IL-6

**Fig. 2:**
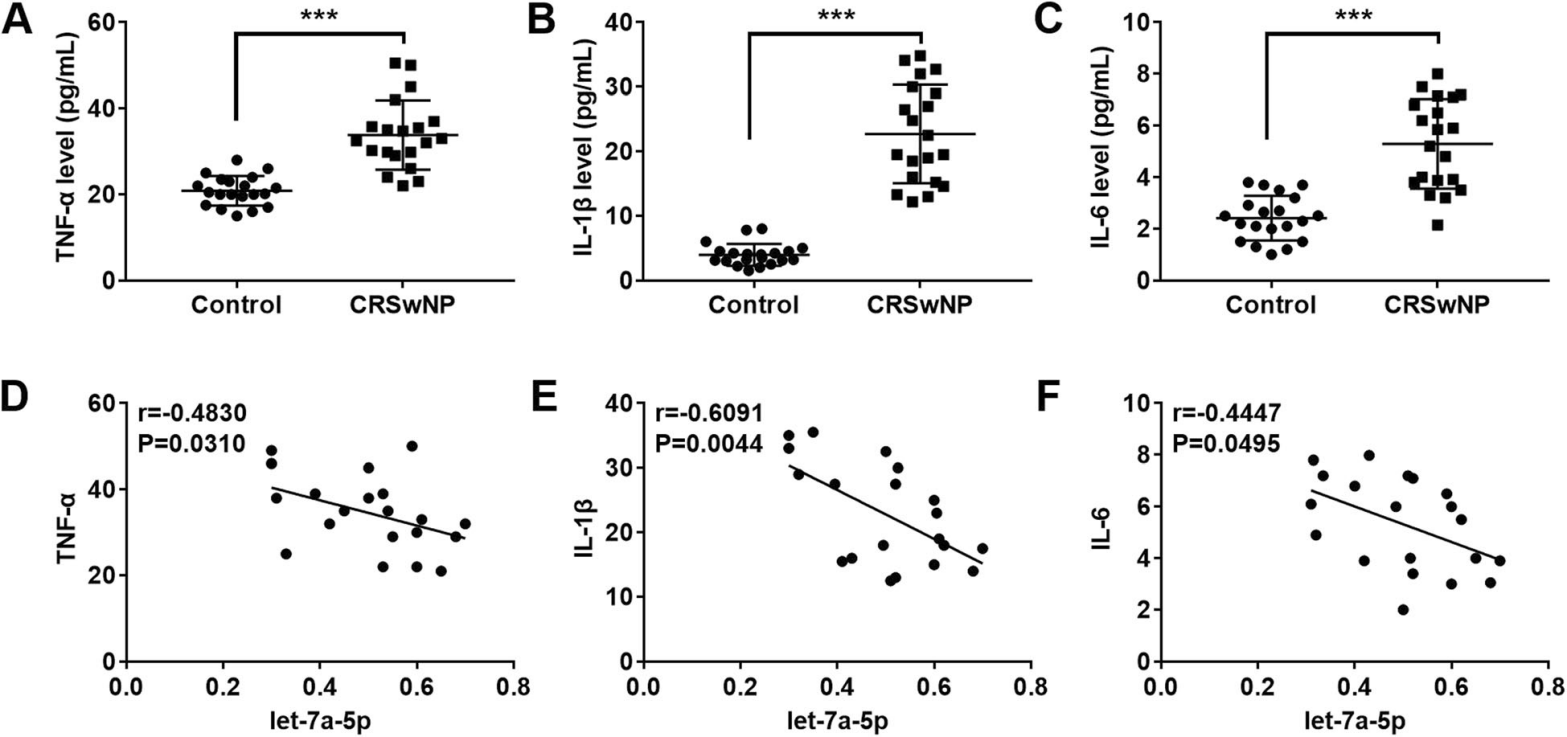
Let-7a-5p was negatively correlated with the inflammatory factors. **a**-**c** The expression level of TNF-α, IL-1β and IL-6 in CRSwNP tissues was detected by ELISA assay. **d**-**f** The correlation between let-7a-5p and inflammatory factors (TNF-α, IL-1β and IL-6) was explored by Spearman’s correlation analysis. ***p < 0.001

To explore the relationship between let-7a-5p and the inflammatory factors, the expression of IL-6, TNF-αand IL-1β was detected by RT-qPCR. The results showed that TNF-α, IL-1βand IL-6 expression were obviously upregulated in CRSwNP tissues compared with the control group (*p* < 0.001; Fig. 2a-c). Next, we detected the correlation between let-7a-5p and the inflammatory factors in CRSwNP tissues by Spearman’s correlation analysis. We noticed that let-7a-5p expression was negatively correlated with TNF-α (*p* = 0.0310; Fig. 2d), IL-1β (*p* = 0.0044; Fig. 2e), and IL-6 (*p* = 0.0495; Fig. 2f) in CRSwNP.

### Let-7a-5p inhibited TNF-α, IL-1βand IL-6 expression in NPDFs cells

**Fig. 3:**
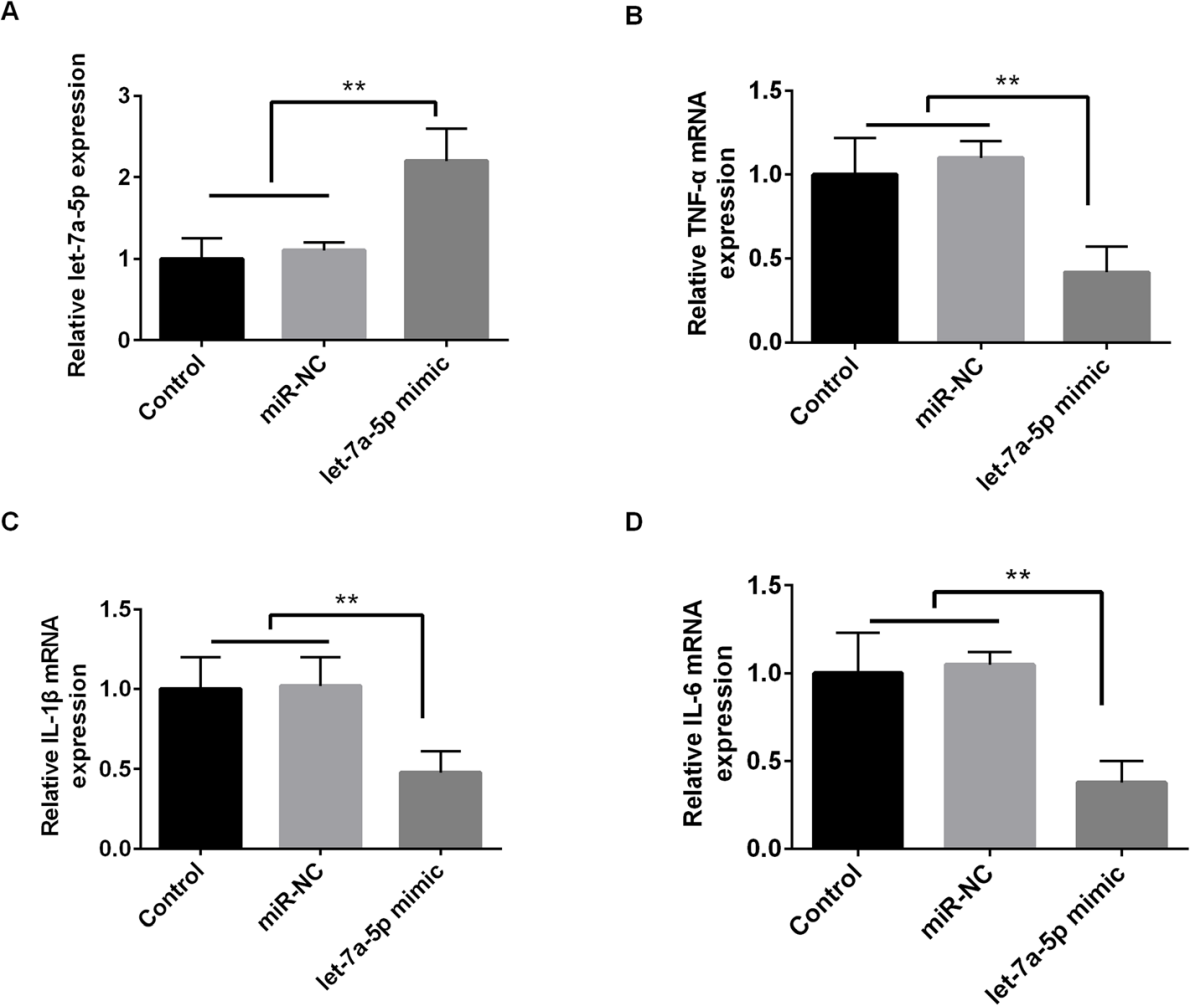
Let-7a-5p inhibited TNF-α, IL-1βand IL-6 expression in NPDFs cells. **a** The expression of let-7a-5p was significantly increased by let-7a-5p mimic. **b**-**d** Let-7a-5p mimic inhibited the mRNA expression of TNF-α, IL-1β and IL-6. **p < 0.01

To explore the specific relationship between let-7a-p and the inflammatory factors in CRSwNP, we transfected let-7a-5p mimic into NPDFs cells. As shown in Fig. 3a, the expression of let-7a-5p was significantly increased by let-7a-5p mimic (*p* < 0.01). Then, we found that let-7a-5p mimic obviously reduced the expression of TNF-α, IL-1β and IL-6 (*p* < 0.01; Fig. 3 b-d). Our findings demonstrated that let-7a-5p overexpression inhibited the inflammatory response in CRSwNP cells.

### Let-7a-5p inhibited the Ras-MAPK pathway in CRSwNP

**Fig. 4:**
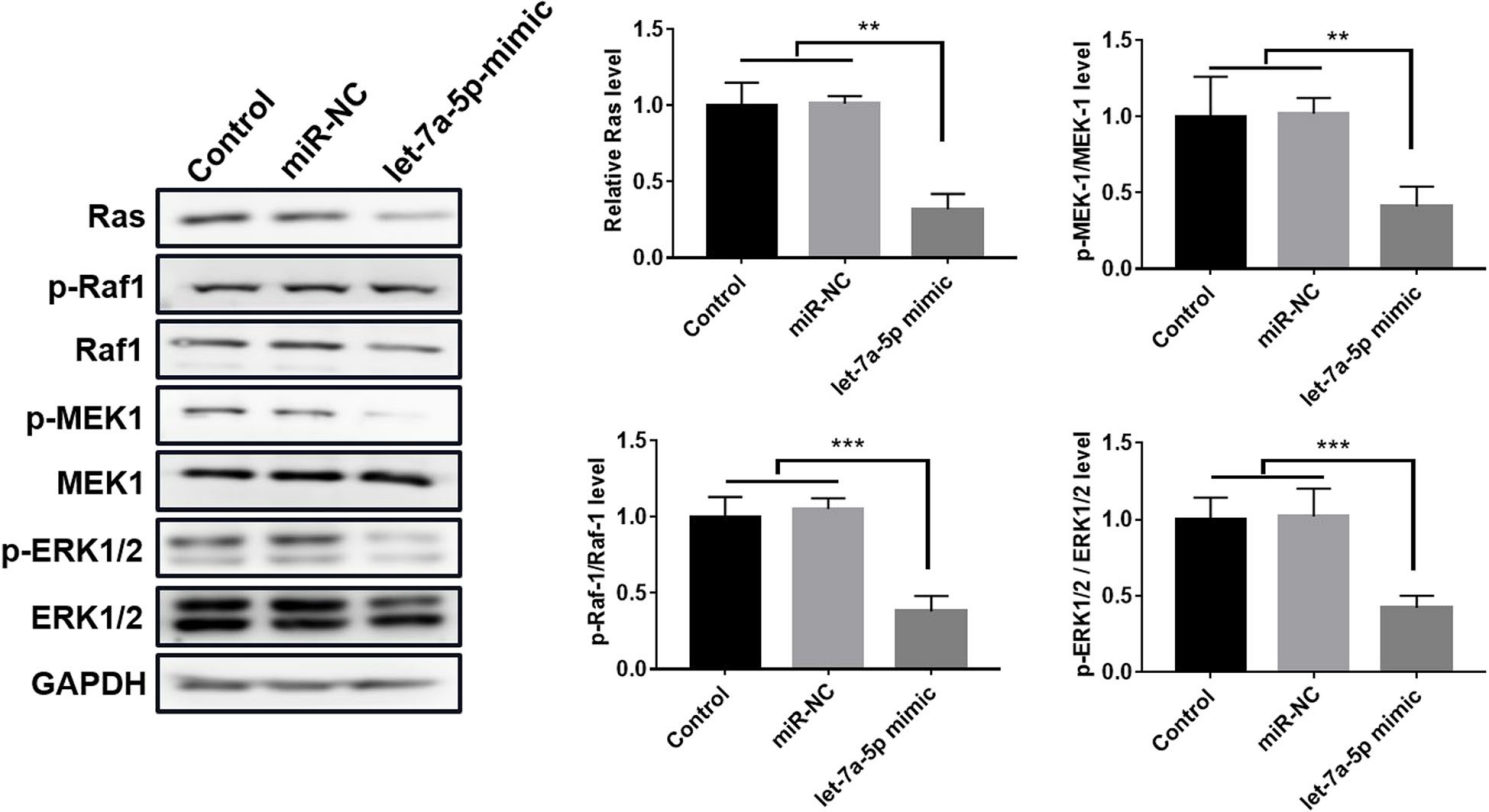
let-7a-5p inhibited the activity of Ras-MAPK pathway in CRSwNP. Western bolt assay was used to explore the protein expression of Ras, Raf1, p-Raf1, MEK1, p-MEK1, ERK1/2 and p-ERK1/2 in NPDFs cells transfected with let-7a-5p mimic. **p < 0.01; ***p < 0.001

Next, we investigated the effect of let-7a-5p on the Ras-MAPK pathway using Western blot assay. The results displayed that let-7-5p mimic markedly reduced the protein levels of Ras, p-Raf1, p-MEK1 and p-ERK1/2, but had no effect on Raf1, MEK1 and ERK1/2 (*p* < 0.01; *p* < 0.001; Fig. 4). These results indicated that let-7a-5p repressed the Ras-MAPK pathway in CRSwNP.

### IL-6 was a downstream target gene of let-7a-5p

**Fig. 5:**
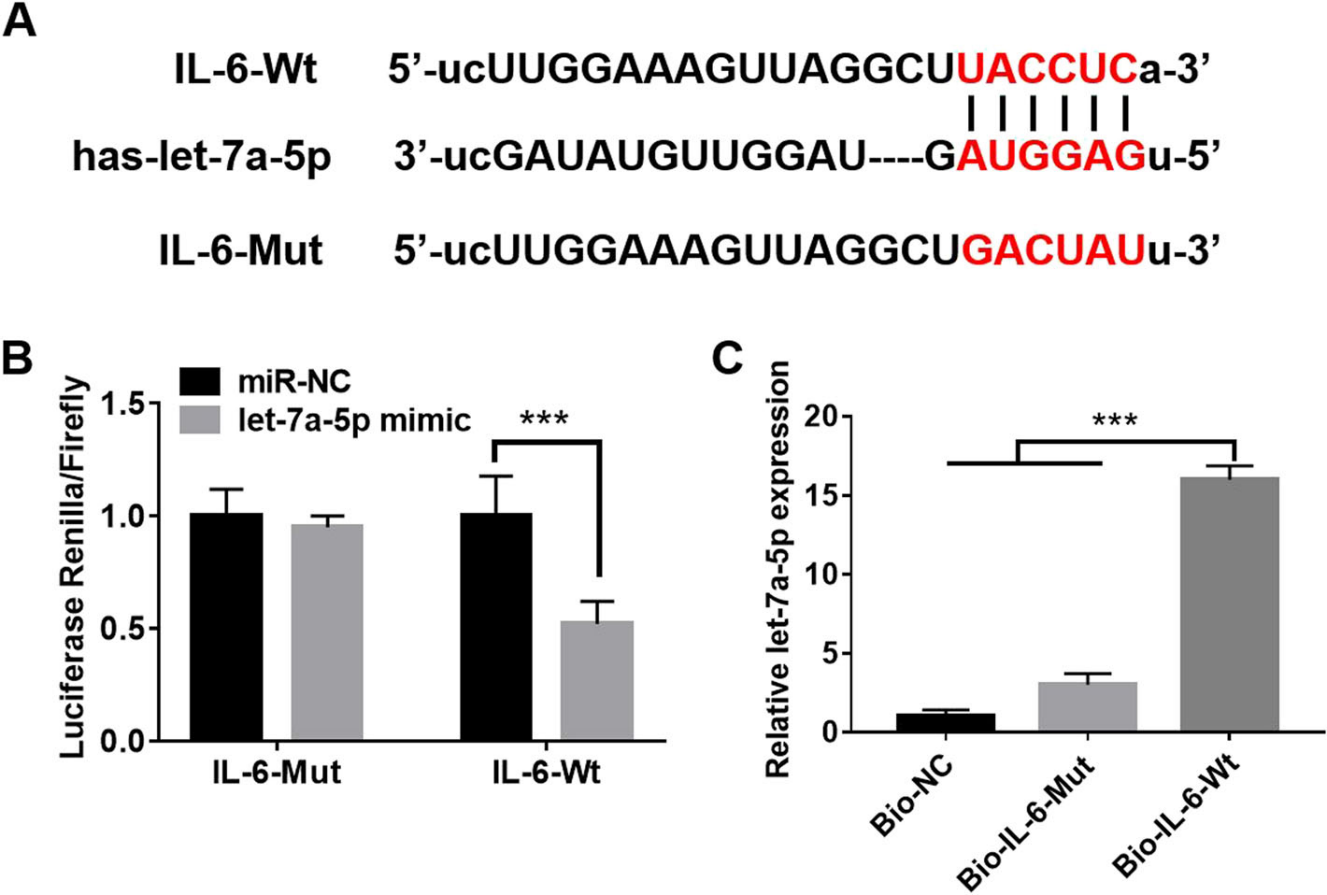
IL-6 was a downstream target gene of let-7a-5p. **a** TargetScan predicted that there were binding sites between let-7a-5p and IL-6. **b** Dual luciferase reporter assay was used to detect the relationship between let-7a-5p and IL-6. **c** RNA pull down assay was used to investigate the binding ability of let-7a-5p and IL-6 in NPDFs cells. ***p < 0.001

As shown in Fig. 5a, there were special binding sites between let-7a-5p and IL-6. To verify this hypothesis, dual luciferase reporter and RNA pull down assay were used to explore the relationship between let-7a-5p and IL-6. We noticed that let-7a-5p mimic significantly reduced the luciferase activity of IL-6-Wt, while had little effect on IL-6-Mut (*p* < 0.001; Fig. 5b). Furthermore, an RNA pull-down assay was used to explore the association between let-7a-5p and IL6. The results displayed that endogenous let-7a-5p was also pulled down by Bio-IL-6-Wt, but not IL-6-Bio-Mut (*p* < 0.001; Fig. 5c). All results indicated that IL-6 was a target gene of let-7a-5p.

### Overexpression of IL-6 eliminated the inhibitory effect of let-7a-5p on inflammatory response in CRSwNP

**Fig. 6:**
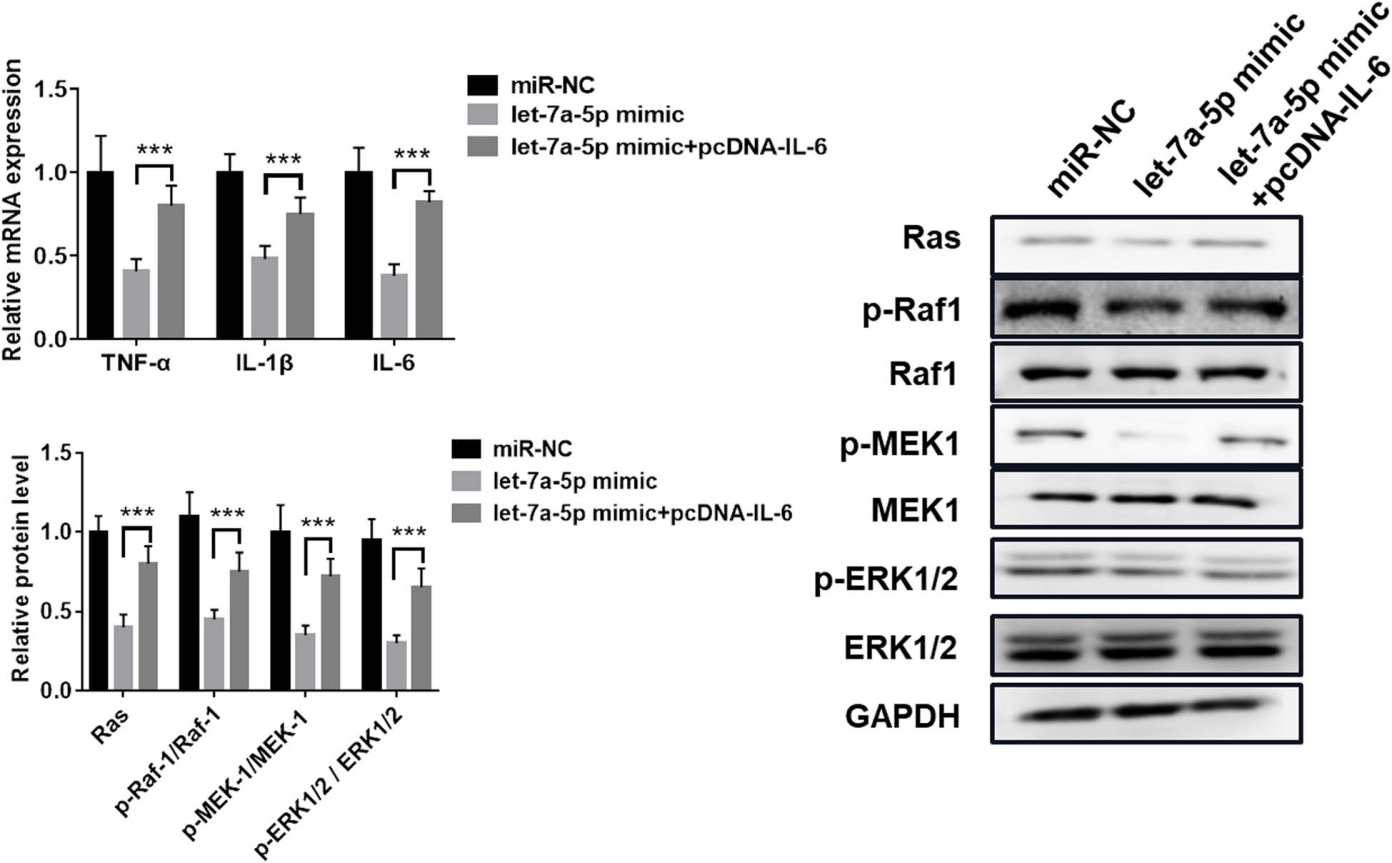
Overexpression of IL-6 eliminated the inhibitory effect of let-7a-5p on inflammatory response in CRSwNP. **a** The mRNA expression level of TNF-α, IL-1β and IL-6 in NPDFs cells with pcDNA-IL-6 and let-7a-5p mimic. **b** The protein expression level of Ras, p-Raf1, Raf1, p-MEK1, MEK1, p-ERK1/2 and ERK1/2 in NPDFs cells with pcDNA-IL-6 and let-7a-5p mimic. ***p < 0.001

To explore the effect of let-7a-5p/IL-6 on the inflammatory response in CRSwNP, we transfected pcDNA-IL-6 into NPDFs cells with let-7a-5p mimic. We noticed that let-7a-5p mimic significantly reduced the expression of TNF-α, IL-1β and IL-6, but IL-6 overexpression reversed the inhibitory effect of let-7a-5p on TNF-α, IL-1β and IL-6 expression (*p* < 0.001; Fig. 6a). In addition, let-7-5p mimic obviously reduced the protein levels of Ras, p-Raf1, p-MEK1 and p-ERK1/2, while IL-6 overexpression destroyed the effect of let-7a-5p (*p* < 0.001; Fig. 6 b). Hence, all data indicated that IL-6 eliminated the inhibitory effect of let-7a-5p on inflammatory response in CRSwNP.

## Discussion

CRSwNP is a heterogeneous disease caused by nasal inflammation and sinus mucosa inflammation. The clinical etiology and pathophysiological mechanism of nasal polyps are complex [16]. Studies performed on CRSwNP mainly focus on exploring potential inflammatory factors and targeted therapy [17, 18]. Moreover, a previous study showed that miR-4492/IL-10 interaction played a crucial role in CRSwNP [19]. Xiao et al. discovered that IL-21 was highly expressed in CRSwNP groups, and suggested that IL-21 might be a therapeutic target for CRSsNP therapy [20]. Our study demonstrated that let-7a-5p was obviously downregulated in CRSwNP tissues and cells. Intriguingly, let-7a-5p was found to be downregulated in nasopharyngeal carcinoma [21]. Next, we found that let-7a-5p inhibited the expression of TNF-α, IL-1β and IL-6, and was negatively correlated with TNF-α, IL-1β and IL-6.

Ras-MAPK pathway has been reported to be involved in the regulation of various multiple cellular processes, such as cell proliferation, differentiation, transcription and apoptosis, and is closely related to inflammation and oxidative stress [22]. In our study, let-7a-5p was found to suppress the Ras-MAPK pathway in CRSwNP. Similar to our findings, Liu et al. verified that NEAT1 regulated the cisplatin resistance by modulating let-7a-5p and the Ras-MAPK pathway in nasopharyngeal carcinoma [21]. On the other hand, KSR1 was found to regulate inflammatory factors’ expression by activating the Ras-MAPK pathway in ischemia/reperfusion injury [23].

IL-6 is a multifunctional, multidirectional cytokine with important physiological and pathological features, and plays an important role in inflammation, immune defense and tissue damage [24]. Tian et al. found that IL-6 was upregulated in CRS, and IL-6 might be involved in the inflammation of CRS [25]. Moreover, Il-6 was proved to be highly expressed in the CRS mucosa, and mediated the accumulation of inflammatory cells and participated in the inflammatory reaction process [26]. In the current work, IL-6 was proved to be a target gene of let-7a-5p. In parallel to previous studies, our data indicated that IL-6 eliminated the inhibitory effect of let-7a-5p on inflammatory response in CRSwNP.

## Conclusion

In general, we found that let-7a-5p was downregulated in CRSwNP. Besides, let-7a-5p was demonstrated to regulate the inflammatory response by interacting with IL-6 through the Ras-MAPK pathway in CRSwNP. We suggested that let-7a-5p might be a possible biomarker for predicting CRSwNP. However, further experiments are needed to verify the effect of let-7a-5p on CRSwNP.

## Data Availability

The datasets used or analyzed during the current study are available from the corresponding author on reasonable request.

## Abbreviations

CRS: Chronic rhinosinusitis
CRSwNP: Chronic rhinosinusitis without nasal polyps
IL-6: Interleukin-6
DMEM: Dulbecco’s Modified Eagle Medium
FBS: Foetal Bovine Serum
EDTA: Ethylene Diamine Tetraacetic Acid
RT-qPCR: Reverse transcription-quantitative PCR
SDS-PAGE: Sodium dodecyl sulfate polyacrylamide gel electrophoresis
